# The Control of Typhoid Infection

**Published:** 1913-01

**Authors:** Charles T. Nesbit

**Affiliations:** Superintendent of Health, Wilmington, N. C.


### THE CONTROL OF TYPHOID INFECTION

CHARLES T. NESBIT, M. D., WILMINGTON, N. C.

*Superintendent of Health*

The typhoid problem in Wilmington as elsewhere is occasioned by the exposure of infected feces to flies, and by the pollution of the soil and water. In rural districts, towns and small cities where the use of the sewer is limited or does not exist, it is obvious that the first attempt at typhoid control must be directed toward the sanitary disposal of human excreta, more especially that of typhoid patients.

In the absence of a system of sewers the sanitary disposal of excreta is not so simple as it would at first appear. All forms of sanitary privy are open to objections and most of these objections are easily magnified by property owners who desire to avoid expense and citizens who are unwilling to complicate their manner of living. The ordinary open surface-privy appeals strongly to the ignorant because all fluids are readily absorbed by the earth and the solids when unconfined rapidly become inoffensive

The sanitary closet, if not cared for properly, soon becomes offensive, and this is a powerful factor in the creation and continuation of the objection to its use.

There were more than five thousand surface-privies in Wilmington at the beginning of June, 1911. The death records of the health office furnished conclusive evidence that typhoid fever had been endemic for a great number of years. An estimate of the number of cases occurring each year made from the number of deaths from this cause shows an average for the five years preceding 1911 of two hundred cases each year, and the time of greatest frequency invariably occurred during the summer months, which indicated fly-borne infection.

The inauguration of the new health department in Wilmington, June 1, 1911, occurred while a rapidly spreading epidemic of typhoid was in progress. Our first efforts were directed to the destruction of the fly which was obviously the means of transmitting this disease. Along with this went the effort to introduce the sanitary privy. All surface-privies, manure-heaps and places in which flies could breed and feed were repeatedly saturated with pyroligneous acid. The result of this procedure was a prompt checking of the spread of the epidemic. This work was done at the expense of the municipality.

The introduction of the sanitary privy was strongly opposed. Property owners objected to the expense and those who made use of the sanitary privy and who neglected its care complained widely of its offensiveness. This opposition was strong enough to interfere with the enforcement of the sanitary closet ordinance to such an extent that at the beginning of the summer of 1912 there were remaining in the city nearly three thousand surface privies. Many of the sanitary privies which had been installed were not made fly-proof as required by the law. This condition of affairs practically insured a repetition of the annual epidemic of typhoid. The council failed to furnish funds for the use of pyroligneous acid and it was certain that no funds would be furnished unless pronounced epidemic conditions appeared. We determined if possible to prevent the recurrence of the epidemic which had been so long a fixture in this community and to that end adopted the following plan:

Each sanitary closet-can, when removed for cleaning, was thoroughly scrubbed and disinfected, and before being replaced in a closet was filled to one-third of its capacity with a disinfecting solution of known typhoid bactericidal efficiency. In addition a circular letter was addressed to every physician in the city requesting him to notify the health office as soon as the symptoms in any suspected patient suggested typhoid, and to use every means in his power to secure the screening of the patient and the disinfection of all excreta. The health office agreed immediately on notification to cooperate with the physician in his efforts to render the case inocuous. To prevent any typhoid excreta from going through the sewers into the Cape Fear River the health office was to supply every household in which there was a case of typhoid with a steel can in which the excreta from the patient might be kept and disinfected.

This plan went into immediate effect and since May 1 it has been in continuous operation. Both physicians and citizens cooperated fully and the results have been extremely gratifying. As soon as a suspected case of typhoid fever is reported a galvanized steel can of 12½ gallons capacity, containing 3 gallons of disinfecting solution and supplied with a tight lid, is sent to the house of the patient. An extra quantity of disinfectant is supplied for the disinfection of bed linen and clothing. Another can is provided, if desired, for use in disinfecting these articles. The can containing the disinfected excreta is placed in an out-building, kept tightly closed at all times, and is removed, cleaned and redisinfected as often as is necessary, without cost to the citizens. New cans are furnished for every exchange in order to prevent the possibility of offense which might be occasioned by the use of a previously used can. As a result of our efforts we recorded during the month of May, 1912, but 6 cases of typhoid fever; in June, 20 cases; in July, 10 cases; in August, 12 cases, and in September, 11 cases. During the same period in 1911, 250 new cases were reported. Recognizing the fact that the excreta of the typhoid case constitute practically the only means of transmitting this disease, and realizing that from the great number of typhoid cases which have occurred in this city each year for many years past, there must be a number of typhoid carriers in this community (it is obvious that our plan of prevention could not reach these), we must conclude from the results that infection from current cases has been prevented to a very gratifying degree.

Typhoid is essentially a disease of the rural districts, small towns, and small cities where human excrement is not immediately conveyed beyond the limits of infection possibility through sewers, and where thorough disinfection of typhoid excreta is least likely to be practiced. The plan we have used cannot be carried out successfully where the population is so concentrated and numerous as in larger cities. Thus the disease and this plan of prevention seem eminently suited to each other.—*Journal A. M A., Jan. 4*, 1913.

